# CRISPR Gene Editing in Lipid Disorders and Atherosclerosis: Mechanisms and Opportunities

**DOI:** 10.3390/metabo11120857

**Published:** 2021-12-09

**Authors:** Harry E. Walker, Manfredi Rizzo, Zlatko Fras, Borut Jug, Maciej Banach, Peter E. Penson

**Affiliations:** 1 School of Biological Sciences, Highfield Campus, University of Southampton, Southampton SO17 1BJ, UK; hewalker9@icloud.com; 2 Department of Health Promotion, Mother and Child Care, Internal Medicine and Medical Specialties (Promise), University of Palermo, 90133 Palermo, Italy; manfredi.rizzo@unipa.it; 3 Centre for Preventive Cardiology, Division of Medicine, University Medical Centre Ljubljana, SI-1525 Ljubljana, Slovenia; zlatko.fras@kclj.si; 4 Medical Faculty, University of Ljubljana, SI-1000 Ljubljana, Slovenia; 5 Department of Vascular Diseases, University Medical Centre Ljubljana, SI-1525 Ljubljana, Slovenia; borut.jug@kclj.si; 6 Department of Preventive Cardiology and Lipidology, Medical University of Lodz, 93338 Lodz, Poland; maciej.banach@icloud.com; 7 Cardiovascular Research Centre, University of Zielona Gora, 65046 Zielona Gora, Poland; 8 School of Pharmacy and Biomolecular Sciences, Liverpool John Moores University, Liverpool L3 3AF, UK; 9 Liverpool Centre for Cardiovascular Science, Liverpool L7 8TX, UK

**Keywords:** CRISPR, atherosclerosis, dyslipidaemia

## Abstract

Elevated circulating concentrations of low-density lipoprotein cholesterol (LDL-C) have been conclusively demonstrated in epidemiological and intervention studies to be causally associated with the development of atherosclerotic cardiovascular disease. Enormous advances in LDL-C reduction have been achieved through the use of statins, and in recent years, through drugs targeting proprotein convertase subtilisin/kexin type 9 (PCSK9), a key regulator of the hepatic LDL-receptor. Existing approaches to PCSK9 targeting have used monoclonal antibodies or RNA interference. Although these approaches do not require daily dosing, as statins do, repeated subcutaneous injections are nevertheless necessary to maintain effectiveness over time. Recent experimental studies suggest that clustered regularly interspaced short palindromic repeats (CRISPR) gene-editing targeted at PCSK9 may represent a promising tool to achieve the elusive goal of a ‘fire and forget’ lifelong approach to LDL-C reduction. This paper will provide an overview of CRISPR technology, with a particular focus on recent studies with relevance to its potential use in atherosclerotic cardiovascular disease.

## 1. Introduction

Elevated circulating concentrations of low-density lipoprotein cholesterol (LDL-C) have been conclusively demonstrated in epidemiological and intervention studies to be causally associated with the development of atherosclerosis and its sequelae, including myocardial infarction, ischaemic stroke, and cardiovascular disease (CVD) mortality [1]. Statins reduce endogenous cholesterol production, resulting in lower LDL-C, and reduce cardiovascular events by approximately one quarter for each year for which they are taken [2]. Cardiovascular risk is closely related to lifetime exposure to LDL-C, and hence long-term reduction of LDL-C is essential, according to the maxim that ‘lower is better for longer’ [3,4,5,6]. Statins are safe, effective, and generally well-tolerated. However, compliance with daily dosing can be problematic for some individuals, resulting in poor clinical outcomes [7]. In particular, statin-associated adverse effects can result in statin intolerance [8,9,10,11], a condition in which an individual is unable to take any statin at all (complete statin intolerance) or is unable to achieve their guideline-directed dose (partial statin intolerance). Whilst compelling evidence suggests that the frequency of statin-associated adverse effects is greatly overestimated [12,13,14,15], this remains a problem in some individuals.

Enormous advances in LDL-C reduction have been achieved in recent years through drugs targeting proprotein convertase subtilisin/kexin type 9 (PCSK9). LDL-C is removed from the circulation via interaction with hepatic LDL-receptors, which mediate endocytosis of LDL particles in the liver, removing them from the circulation. Inside the cell, the LDL receptors dissociate from the LDL particles and are recycled to the cell membrane [16]. Once removed from the circulating blood, LDL particles are unable to be deposited in the blood vessel walls and are therefore unable to cause the manifestations of atherosclerosis. Upregulation of hepatocyte LDL receptors is a common feature of lipid-lowering drugs and non-pharmacological interventions which reduce adverse cardiovascular outcomes [16]. PCSK9 is a regulatory molecule that causes the internalization and degradation of the LDL receptor and thus reduces hepatic LDL uptake. Monoclonal antibodies directed against PCSK9 result in increased expression of hepatic LDL-receptors, lower circulating LDL-C and reduced CV events [17]. These monoclonal antibodies are delivered via subcutaneous injection and can achieve their therapeutic effects with dosing intervals of two-to-four weeks. More recently, inclisiran was developed; it reduces the production of PCSK9 through messenger ribonucleic acid (mRNA) silencing. Twice-yearly administration of inclisiran reduces LDL-C by over 50% in a range of patient groups, with only mild adverse effects, and the effect of inclisiran on cardiovascular outcomes is currently being evaluated in large randomized controlled trials [18,19,20,21]. Finally, there are also attempts on the anti-PCSK9 vaccine to ensure endogenous generation of monoclonal antibodies against PCSK9 protein and ensure effective continuous reduction of LDL-C [22,23,24].

The monoclonal antibody inhibitors of PCSK9 and inclisiran are remarkable for their efficacy and safety and provide enormous advances over existing therapy [25,26,27]. However, both require repeated dosing throughout the life course. In the long term, therefore, it would be attractive to develop ‘fire and forget’ strategies, whereby a potential single one-off intervention could be used to reduce an individual’s LDL-C for the rest of their life. Such approaches perhaps have the greatest chance of success when they mimic the effects of naturally-occurring mutations and polymorphisms, which are known to have a beneficial effect on lipid profiles. The identification of PCSK9 as a target for lipid-lowering drugs occurred as a result of the recognition that loss-of-function mutations of PCSK9 resulted in reduced circulating concentrations of LDL-C and a lower risk of cardiovascular disease [28]. Similarly, promising therapeutic advances have resulted from the observations that loss-of-function mutations of the angiopoietin-like 3 gene (ANGPTL3) result in decreased LDL-C and triglycerides [29]; and that loss-of-function mutations of the gene encoding apolipoprotein C3 (ApoC3) result in lower plasma triglycerides and reduced risk of coronary heart disease [30].

Recent experimental studies suggest clustered regularly interspaced short palindromic repeats (CRISPR) gene-editing targeted at PCSK9 may represent a promising tool to achieve this elusive goal [31]. This paper will provide an overview of CRISPR technology, with a particular focus on recent studies with relevance to its potential use in atherosclerotic cardiovascular disease (ASCVD).

## 2. CRISPR Gene Editing

### 2.1. CRISPR in Bacterial Adaptive Immunity

**Figure 1:**
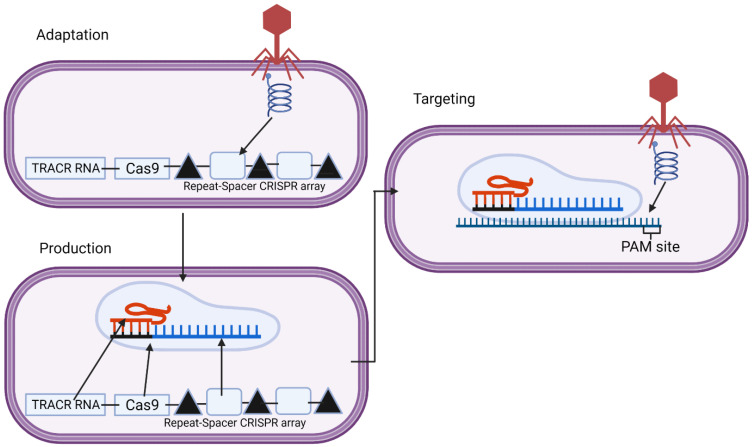
This is the three-stage process of how clustered regularly interspaced short palindromic repeats (CRISPR) works naturally against bacteriophages, with this diagram showing the type 2 system. The adaptation step occurs during the initial infection. A segment of the genome (spacer) is taken and put into the CRISPR array between two repeats. Upon future infection, the array is transcribed, forming the cRNA along with the trans-activating CRISPR RNA (tracrRNA) and the Cas genes, in this case, Cas9. These then come together to form a complex, which then binds to the region of the bacteriophage genome complementary to the spacer, and Cas9 cleaves the genome. Created with BioRender.com.

Bacteria constantly thwart bacteriophage infections, utilizing many innate non-specific immune defences, but also CRISPR, a system of adaptive immunity, which is far more specific. CRISPR uses DNA fragments from previous bacteriophage infections to detect and destroy DNA from similar bacteriophages during later infections (Figure 1). There are two classes of CRISPR systems with six types across these two classes utilized by different bacteria, with types I, II and III being the most important [32]. CRISPR requires two parts—a guide RNA and Cas proteins. A guide RNA is necessary for sequence-specific targeting, whereas Cas proteins have structural roles, and some of them also have nuclease domains, which are responsible for cleaving the DNA. The RNA binds to a Cas protein to successfully target and cut the genome. Different systems utilize different Cas proteins and arrangements of these proteins, ranging from Cas1 to Cas13, all of which are utilized in different CRISPR systems [33].

The targeting of bacteriophages through CRISPR is a three-stage process of adaptation, cRNA maturation and interference. During a bacteriophage infection, the bacteria will take a fragment of the bacteriophage genome. This is achieved using a Cas1–Cas2 complex, which is highly conserved in many CRISPR systems, which may also include other proteins depending on the system such as Cas4 in some systems, including type I-A, B and C and type II-B and Cas9 with tracrRNA and Csn2 in type II-A systems, with many other proteins also implicated in adaptation across different CRISPR systems [34,35,36,37]. This complex searches the bacteriophage genome looking for specific features, such as a protospacer adjacent motif (PAM) site adjacent to a target sequence, as this is necessary for CRISPR to target it. Cas9 ensures there is a PAM site adjacent to the target site in type II-A systems [36]. The PAM site is necessary to enable selectivity so that the CRISPR does not cut its own genome at the spacer [38]. When a suitable region is identified, this complex cuts out the region through the nuclease activity of Cas1, which is then incorporated into the CRISPR repeat-spacer array [39]. In this array, there are spacers from the different bacteriophages the bacteria has been infected by in the past; these spacers are flanked by repeat regions with a leader region before the first repeat. Spacer integration happens at the leader proximal end so that the newer infections, closer to the leader, are more strain-specific, whilst the older spacers, which are less specific, may be lost [40]. These spacers are incorporated through a transesterification reaction where the 3’ hydroxyl ends of the spacer attack the top strand leader-repeat junction before attacking the bottom strand leader-spacer junction incorporating the spacer into the array [41]. The first repeat duplicates to get the two flanking repeats of the spacer [42]. The Cas1–Cas2 complex is also important here, guiding the spacers to the site for integration, but how it does this varies across different CRISPR systems [41].

Upon future infection, the CRISPR array and Cas genes are transcribed. Transcription of these genes is activated and repressed through promoters and regulatory elements within the leader sequence binding regulatory proteins and other signals. These signals vary across CRISPR systems but include elements such as LeuO and cAMP receptor protein, which are both associated with phage infection [42]. Upon activation of the array, the spacers and flanking repeats are transcribed to form the pre-cRNA, which is then processed to form the mature cRNA by Cas proteins and ribonucleases [33]. In systems I and III, this mature cRNA includes part of the repeat regions, which can form stem-loops which are important for the binding and activation of the Cas proteins [42]. The RNA forms these secondary structures before binding a complex containing 5 or 6 different Cas proteins; these are known as the cascade in type I and the Csm or Cmr complex in type III [37]. The type II system is different as the repeat regions cannot form stem-loops; instead, another RNA—called the trans-activating RNA—is used, which is complementary to these repeat regions, binding the repeat regions and recruiting RNase 3 and Csn1 for the maturation of the cRNA [43].

The cRNA is complementary to the specific phage genome, which has currently infected the bacteria; it will thus bind the complementary region of the genome at the PAM site with the associated Cas protein. Each CRISPR system includes a Cas protein with nuclease function for the interference step—type I uses Cas3, type III uses Cas10 and Cmr4 (one of the Cas7 family), and type II uses Cas9 [33,44]. These proteins will cleave the genome forming a double-stranded DNA break (in the case of type I and II) or an RNA break (in the case of type III), thus preventing bacteriophage replication.

### 2.2. CRISPR as a Potential Technology for Gene Therapy

**Figure 2:**
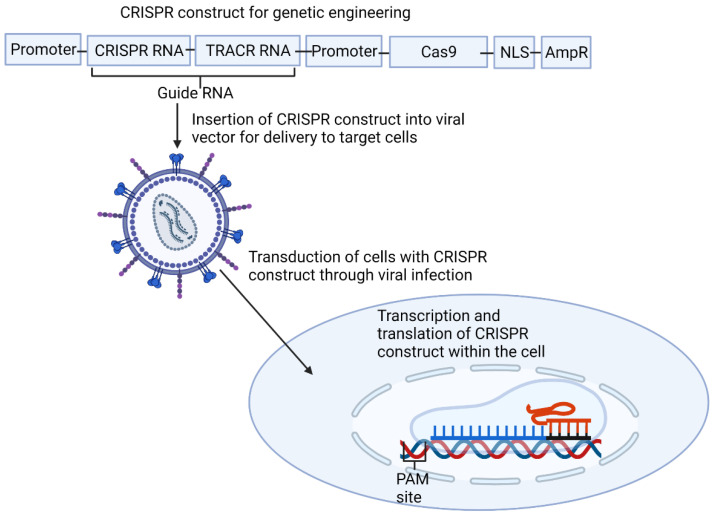
This shows how the type 2 clustered regularly interspaced short palindromic repeats (CRISPR) system is utilized for genetic engineering. Firstly, a construct is made containing all the necessary CRISPR elements, including a guide RNA designed to be complementary to the target region. This is then cloned into a plasmid before being inserted into a viral vector. This vector infects the target cells, transducing the cells with the CRISPR construct, which is then transcribed and translated before forming the complex and cleaving the region of the genome it was designed to target. AmpR, Ampiciline resistance promoter; NLS, nucleic localization signal; TRACR trans-activating CRISPR. Created with BioRender.com.

CRISPR is now being utilized in gene therapy and genetic engineering. It has clear advantages over other approaches, such as zinc finger nucleases and transcriptor activator-like effector nucleases (TALENs)—it is easier to target and produce due to it being RNA-based rather than protein-based, and has the ability to target larger regions of the genome through multiplexing, where multiple CRISPR–Cas9 complexes are targeted to adjacent regions of the genome [45] (Figure 2). The type II system has been utilized for gene therapy because it only requires one Cas protein for interference. In contrast, Class 1 CRISPR systems require multiple Cas proteins, which presents greater challenges in delivery to the nucleus of the cells.

The process of using CRISPR for gene therapy starts by identifying a target gene and a coding region within it, which has an adjacent PAM site so it can be targeted. After designing a cRNA complementary to a part of this coding region, it is cloned into a plasmid also containing a Cas9 gene and the trans-activating CRISPR RNA (tracrRNA) sequence. Promoters are also needed for the transcription of the Cas9 gene and the guide RNA—the combination of the cRNA and the tracrRNA [46]. As Cas9 is a bacterial protein, nuclear localization sequence (NLS) is included at the end of Cas9 to target this to the nucleus [47]. An antibiotic resistance gene is also included to provide a selection marker for whether cells have successfully taken up the plasmid. Once the cRNA has been inserted into the plasmid, it is cloned into a viral vector using a packaging cell line. Once in this viral vector, the target cells are infected with the virus, transducing the cells with the CRISPR construct, which localizes to the nucleus, before being transcribed and leaving the nucleus for the Cas9 transcript to be translated. The CRISPR-Cas9 complex then forms and enters the nucleus again, binds to the target gene through the sequence specificity of the guide RNA and Cas9 then cleaves both strands of DNA through its two nuclease domains—HNH and RuvC, forming a double-stranded DNA break [48]. There are then two options for repair: either non-homologous end-joining (NHEJ) or homology-directed repair (HDR). NHEJ modifies the ends before ligating the broken strands back together with no regard for homology, leading to frequent indel mutations. HDR is a far more accurate process but is used far less frequently, using a homologous template, often from a sister chromatid, which is copied, ensuring the repair is correct [49]. Rates of HDR can be improved by adding in a template for the sequence to be repaired with the CRISPR complex [50]. Adding a template with HDR also allows the insertion of a sequence into a gene, known as a knock-in, through including a sequence to be inserted in the template or the correction of mutations through changing the base in the template to correct the mutation when this is copied into the genome [50].

### 2.3. Problems and Issues with CRISPR in Genetic Engineering

There are two prominent issues with using CRISPR-Cas9 for genetic engineering—non-specific targeting and poor rates of success in vivo. The non-specific targeting is due to the relatively short sequences used for CRISPR—around 20 nucleotides long—being prone to non-specific targeting. This means the guides used for CRISPR may bind sequences that are not the target sequence but have a similar homology, with up to 6 mismatches [51]. When Cas9 cleaves these sequences, if the repair is incorrect, which is likely with NHEJ or if an exogenous template is added for HDR, mutations may be introduced and disrupt the function of other genes, possibly leading to cancer if appearing in an oncogene. The guides binding to these mismatched sequences is far more likely if the mismatch is at the other end of the sequence to the PAM with the first 12 bases of the guide forming the CRISPR seed sequence, which must be complementary to the target sequence for successful targeting. The guide will also not bind to the mismatch if it is between bases 4 and 7 of the guide as this core region has no tolerance of these mismatches [51]. Other gene-editing systems utilize longer stretches of nucleotides, such as meganucleases, which recognize stretches of up to 40 nucleotides. However, these are hard to target because it is hard to change the sequence they recognize [52]. There are further problems regarding the repair methods with HDR happening rarely when a correct fragment is not provided, at around 20%, with cells favouring NHEJ, and NHEJ is still used more often when a donor template is included with the CRISPR complex for HDR [49].

The problem of poor success rates in vivo is not due to an issue with CRISPR itself but rather the delivery of the CRISPR construct. Vectors are currently being designed to deliver this CRISPR construct to cells, and this has great success in vitro, as demonstrated by Jennifer Doudna and Emmanuelle Charpentier, who were awarded the 2020 Nobel prize in chemistry for their achievement [53]. However, when conducted in vivo, rates of success are far lower due to the large mass of humans, meaning large doses are required to get a high enough transformation efficiency to have a good therapeutic effect through intravenous injection [54]. Other methods of giving CRISPR therapies may require lower doses but may be significantly more intrusive or untranslatable from the mouse models they have been tested on [54]. There are also issues with targeting vectors for tissue-specific therapeutic effect with one of the more popular vector choices, adeno-associated viruses, restricted to targeting a few organs due to the viruses limited natural tropism. It is possible to engineer the vector to expand its tropism to target more tissues or use vectors with a naturally larger tropism [54]. The final issue faced with adeno-associated viruses is the size they are able to carry with adenoviral vectors having a capacity of around 4.7 Kb, whilst the Cas9 gene alone is 4.2 Kb, leaving limited room for the other necessary components. However, adenoviral vectors easily have enough capacity to provide a useful alternative [55,56]. Other solutions for this include using a truncated version of Cas9 or putting the Cas9 and the single guide RNA in two separate vectors, which will come together again when in the target cells [57,58]. There are many solutions used to help solve these issues, with CRISPR already being used in vivo in a clinical trial to treat a condition called Leber congenital amaurosis that causes blindness [59].

## 3. CRISPR Gene Editing of PCSK9

As described above, long-lasting knockdown of PCSK9 would be expected to reduce circulating LDL-C and thus reduce the manifestations of ASCVD. Specific approaches to achieve this aim using CRISPR and the results of preclinical experiments are described below.

### 3.1. Specific Mechanisms

CRISPR systems can be engineered to perform specific functions, which differ from their natural behaviour. These systems require a partially or completely inactive Cas9, where one or both of the nuclease domains of Cas9 are inactivated, thus removing the nuclease function, so it no longer cleaves both strands of the DNA but still binds with guide RNA. In these engineered systems, the Cas9 is bound to another protein, which then performs its own function, modifying the genome in a different way to how CRISPR usually would. There are three different examples of these modified CRISPR systems, including transcriptional editors, prime editors and base editors (Table 1) [60].

Transcriptional editors use a completely inactivated Cas9 referred to as a dead Cas9, complexed with a transcriptional activator or repressor to introduce an epigenetic modification that will activate or repress transcription, changing the levels of gene expression. These can include methylases to repress transcription and acetylases to activate transcription [61].

Through this mechanism, transition and transversion mutations can be corrected by changing the base that is mutated in the genome in the prime editing guide RNA, which then replaces the mutated strand and can also repair indels through adding or removing a base/bases to or from the prime editing guide RNA that has/have been inserted or deleted from the genome which is then copied in by reverse transcriptase [62].

Base editors introduce precise single-base substitutions. A nickase Cas9 is complexed with a base deaminase, either for cytosine or adenine. This Cas9-base deaminase complex is bound to a guide RNA which then binds the genome, and instead of Cas9 cleaving the DNA, the deaminase chemically modifies either cytosine or adenine, depending on the deaminase being used, leading to C-T mutations or A-G mutations. The other strand is then cleaved and repaired to match this edit. Due to the base editors available-cytosine and adenosine deaminases, only transition mutations can be repaired using base editors, so they are more limited in this aspect than prime editors [31,62].

### 3.2. Experimental Evidence of Proof-of-Concept

In a recent study looking into the potential use of CRISPR to target PCSK9, base editors were used [31]. The chosen targets within the PCSK9 gene were splice donor or acceptor sites at exon-intron boundaries within the gene to disrupt splicing so dysfunctional PCSK9 would be produced and rapidly degraded through nonsense-mediated decay. A total of 20 different base-pair long-guide RNAs were used, each binding at an exon-intron boundary with a splice donor or acceptor with an adjacent PAM site. The CRISPR system employed used an adenine base editor that has a working range of bases three to nine of the guide RNA. The guides needed to bind to an adenine within these bases to enable the base editor to work. The CRISPR construct used contained the guide RNA and the mRNA of an adenine base editor, which was comprised of a nickase Cas9, and a deoxyadenosine deaminase domain. Instead of viral vectors, this study used lipid nanoparticles (LNPs) as the delivery system. LNPs are predominantly taken up by the liver and are thus particularly suitable in the context of targetting PCSK9. The lipid nanoparticles were evaluated at a range of concentrations in three different experimental models [31].

Firstly, the nanoparticles were evaluated in human hepatocyte cells in vitro. When tested, levels of splice site editing were over 60%, and levels of PCSK9 had fallen by 55%—a significant decrease, consistent with what was expected when dysfunctional PCSK9 underwent nonsense-mediated decay [31]. Next, the nanoparticles were evaluated in mice. LNPs were delivered through intravenous infusion and achieved even better editing, with 70% of the base editing at the splice site. The latter suggests that saturation of the editing of the hepatocytes has occurred, as hepatocytes comprise 70% of all liver cells [31]. Finally, the nanoparticles were evaluated in cynomolgus monkeys in vivo. The intervention achieved a mean 63% editing at the splice site, a mean decrease of 81% in PCSK9 levels, and a mean decrease of 65% in LDL-C levels across the different doses (Table 2). This large decrease in PCSK9 levels suggests a similar saturation of hepatocyte editing to that is seen when testing in mice. All the doses used achieved over 50% editing. No adverse health events were seen aside from mild rises in ALT and AST liver enzymes. Liver enzyme activity went back to normal after two weeks, along with the depletion of LNPs levels after two weeks and the depletion of mRNA of the CRISPR complex after one week. Off-target editing was observed in the cynomolgus monkeys at one site at levels of a mean of below 1% across different doses; however, this site shares very little homology to the human genome, so it may not be subject to off-target editing in humans (to be finally confirmed in human studies). This is supported by the fact that there was no off-target editing observed in human hepatocytes [31].

This LDL-reduction observed in the monkey model was equivalent to or greater than that expected with lipid-lowering therapies, such as statins or novel therapeutic interventions, including antibodies and antisense oligonucleotides. Indeed, were a sustained 65% reduction in LDL-C achievable in humans with CRISPR therapy, it would be expected to result in a substantial reduction in cardiovascular events in treated patients. A meganuclease-based therapy has also been investigated, although this achieved far lower levels of splice site editing and higher levels of off-target editing, including in human hepatocytes [63].

### 3.3. Other Potential Uses of CRISPR in Atherosclerosis and Cardiovascular Disease

Whilst the demonstration of successful in vivo CRISPR targeting of PCSK9 in Cynomolgus monkeys represents the most advanced and promising approach to the use of CRISPR in the management of lipids and atherosclerosis, other approaches and targets are also of great interest as potential therapeutic strategies. Increasing interest in the research community with respect to CRISPR has followed the successful demonstration that a single dose of CRISPR-Cas9 nanoparticles could cause the robust and persistent reduction in the mouse hepatic transthyretin gene [64]. This finding paved the way for lipid-modifying approaches targeting the liver. In an attempt to replicate the beneficial effects associated with loss-of-function mutations of ApoC3 (lower plasma triglycerides and reduced risk of coronary heart disease) [30], CRISPR/Cas9 has recently been used to inactivate ApoC3 in Syrian golden hamsters, which have a similar metabolic profile to humans [65]. The investigators found that inactivation of ApoC3 led to improved lipid profile (particularly reduced triglycerides) in hamsters receiving a high-fat, high-cholesterol diet and led to the development of fewer atherosclerotic lesions [48]. If such an approach could be replicated in humans in vivo, it could provide an important additional tool to aid in the management of residual risk factors for cardiovascular disease, particularly in patients at high risk of events, for whom LDL-C is well-controlled on current lipid-lowering therapy [3,4,66,67,68,69]. Recent experiments suggest that CRISPR approaches to treating hypertension [70] and arrhythmias [71,72] may also be possible, broadening the potential scope of this technology. Looking further into the future of research and development, CRISPR technologies have proven to be useful experimental tools in identifying new targets for the development of therapeutic agents. Whilst, therapeutic approaches to the use of CRISPR inevitably involve the treatment of fully developed organisms, CRISPR/Cas9 can also be injected into fertilized oocytes, effectively producing knockout animals [73]. This approach has greatly expanded the range of available knockout animals available for lipid research, including rats and pigs with ApoE and LDL-receptor knockouts [73,74,75].

## 4. Conclusions and Future Directions

CRISPR therapy looks very promising, proving very effective at editing the PCSK9 gene and reducing levels of LDL cholesterol with very few side effects in experimental and preclinical models. Clearly, trials of entirely novel therapeutics in humans will require great caution and careful surveillance to enable early identification of any long-term undesirable effects and to evaluate the long-term efficacy in terms of plasma lipids, markers of atherosclerosis and cardiovascular events. Nevertheless, a successful CRISPR approach to PCSK9 targeting would provide an important ‘proof-of-concept’ and would have implications in cardiovascular therapeutics beyond the expected anti-atherosclerotic effects. Clearly, the implications for using such multiple gene-editing therapies and other biological drugs in a single individual will need scientific, clinical, ethical and broader societal addressing in the future. However, for now, we can say that CRISPR has the potential to reduce the ASCVD burden, and we await future data with great interest.

## Figures and Tables

**Table 1:** Summary of Type 2 CRISPR system tools.

CRISPR System	Identification	Formation and Parts	DNA Binding	Action Upon Binding DNA	Ref.
Natural	A target site in the bacteriophage genome is identified through a Cas1–Cas2 complex which binds the invading viral DNA and takes a region that it then integrates into the CRISPR array, ensuring a PAM sequence adjacent to this site is present before choosing this spacer. Different subtypes of type II systems require other proteins, such as II-A needs Cas9 and Csn2 and II-B needs Cas4.	Upon future bacteriophage infection, the CRISPR array is transcribed, transcribing the spacers from past infections. These then complex with Cas9 and the tracrRNA, forming the CRISPR complex.	The correct spacer for the current infection and Cas9 will then bind the viral DNA at the site. The spacer is specific and complementary to Cas9, recognizing the PAM sequence, as is required for binding.	The Cas9 protein, specifically the RuvC and HnH domains, cleave the double-stranded DNA genome of the bacteriophage, stopping the infection.	[33,36,38]
Engineered	A disease is identified through the phenotype shown with the disease before studying the genome to find an appropriate target gene and a target site within this gene with an adjacent PAM sequence which a guide RNA can be designed against for targeting.	The tracrRNA and spacer are merged to form a guide RNA. This is then put in a vector with the Cas9 gene, which is delivered to cells and the guide RNA and Cas9 are expressed, forming the CRISPR complex.	The guide RNA is designed to be complementary to the target DNA, which is causing a disease, so it will bind at the target site with Cas9, which recognizes the adjacent PAM sequence, allowing binding so that Cas9 can then cleave the DNA.	The RuvC and HnH domains of Cas9 cleave double-stranded DNA, which is then repaired either through NHEJ or HDR. HDR can be used with an exogenous template to insert a sequence into the genome and correct mutations.	[38,46,48,50]
Transcriptional editors	The aberrant phenotype shown is used to identify the disease before analyzing the genome to find the gene responsible, and a guide RNA is then designed against a site within this gene with an adjacent PAM sequence to change the gene expression through epigenetic modification.	A guide RNA is put in a vector with s-gene coding for the deactivated Cas9 fused to a transcriptional activator or repressor. This is then expressed in the cell, forming the complex.	The guide RNA and Cas9 bind the DNA through Cas9, recognizing the PAM sequence allowing the guide RNA to bind the region it is targeted against. Here Cas9 binds, not for cleavage, as it is deactivated, but for the attached transcriptional control genes to function.	Upon binding the DNA, the transcriptional control gene fused to Cas9 will perform its function by either introducing or removing an epigenetic modification such as a methyl group, which will increase or decrease the gene expression, depending on the modification made.	[38,46,60]
Base editors	The phenotype shown is used to identify the disease before analyzing the genome to find the mutation responsible, and a guide RNA is designed against the mutated site. An adjacent PAM sequence is still required for binding, so some mutated sites may not be possible to target if there is no PAM sequence present. These can only correct transition mutations, not transversion mutations or indels.	Base editors contain a guide RNA with a partially inactivated Cas9 (Cas9 nickase) to cleave one strand. They also have a base editor—either a cytosine or adenosine deaminase fused tthis Cas9 nickase. This is all included in a vector which is then expressed in the cell where the different elements will come together to form the complex.	The guide RNA binds as it does in engineered systems with Cas9 nickase recognizing the PAM site and the guide RNA binding to its target region. Cas9 nickase will only nick one strand whilst the deaminase associated with it acts on the other strand.	Either cytosine or adenine is deaminated depending on which deaminase is being used, to go to uracil or guanine, respectively. Cas9 nickase will nick the other strand, which are then repaired to match the changed base.	[38,46,60]
Prime editors	Identification is carried out in the same way as for base editors, including ensuring the mutated site has an adjacent PAM sequence, but when the guide RNA sequence is made, a correct sequence to be copied in, in place of the mutated sequence, is included. This method means both transition and transversion mutations can be corrected. Indels can be repaired using this method through the inclusion or removal of a base/bases in the prime editing guide RNA that has/have been inserted or deleted in the mutated genome.	Prime editors contain Cas9 nickase, which will cleave one strand fused to reverse transcriptase and a prime editing guide RNA made up of a normal guide RNA, a reverse transcriptase primer binding site, and a sequence to be copied in. This is all put in a vector which is expressed in cells forming the CRISPR complex.	Cas9 nickase recognizes the PAM sequence with the prime editing guide RNA binding the target sequence. Reverse transcriptase binds with Cas9 as it is fused to it.	Firstly, Cas9 nickase nicks one strand. The prime editing guide RNA used in prime editors is longer, containing a sequence that is copied into the genome by reverse transcriptase to correct a mutation, with the original mutated strand replaced by this new edited strand. The other strand is then cleaved and repaired to match the edited strand.	[38,46,60,62]

**Table 2:** Impact of CRISPR base editing on parameters related to atherosclerosis table generated from data in ref. [31].

Parameter	Model	Number of Experimental Animals	Effect
Splice site editing	Human hepatocytes in vitro	NA	>60% splice site editing achieved at splice site
PCSK9 expression	Human hepatocytes in vitro	NA	PCSK9 expression reduced by 55%
Splice site editing	Mouse in vivo	4–5	A 70% liver base editing of the splice site
Splice site editing	Cynomolgus monkeys in vivo	2–3	A 63% base-editing frequency of the PCSK9 splice-site after two weeks
PCSK9 expression	Cynomolgus monkeys in vivo	2–3	A 81% reduction in blood PCSK9
LDL-C	Cynomolgus monkeys in vivo	2–3	A 65% reduction in blood LDL-C

## References

[1] Ference B.A., Ginsberg H.N., Graham I., Ray K.K., Packard C.J., Bruckert E., Hegele R.A., Krauss R.M., Raal F.J., Schunkert H. (2017). Low-density lipoproteins cause atherosclerotic cardiovascular disease. 1. Evidence from genetic, epidemiologic, and clinical studies. A consensus statement from the European Atherosclerosis Society Consensus Panel. Eur. Heart J..

[2] Collins R., Reith C., Emberson J., Armitage J., Baigent C., Blackwell L., Blumenthal R., Danesh J., Smith G.D., DeMets D. (2016). Interpretation of the evidence for the efficacy and safety of statin therapy. Lancet.

[3] Cybulska B., Kłosiewicz-Latoszek L., Penson P.E., Nabavi S.M., Lavie C.J., Banach M. (2020). How much should LDL cholesterol be lowered in secondary prevention? Clinical efficacy and safety in the era of PCSK9 inhibitors. Prog. Cardiovasc. Dis..

[4] Penson P.E., Pirro M., Banach M. (2020). LDL-C: Lower is better for longer—even at low risk. BMC Med..

[5] Banach M., Penson P.E., Vrablik M., Bunc M., Dyrbus K., Fedacko J., Gaita D., Gierlotka M., Jarai Z., Magda S.L. (2021). Optimal use of lipid-lowering therapy after acute coronary syndromes: A Position Paper endorsed by the International Lipid Expert Panel (ILEP). Pharmacol. Res..

[6] Banach M., Penson P.E. (2021). Lipid-lowering therapies: Better together. Atherosclerosis.

[7] Serban M.-C., Colantonio L., Manthripragada A.D., Monda K.L., Bittner V.A., Banach M., Chen L., Huang L., Dent R., Kent S.T. (2017). Statin Intolerance and Risk of Coronary Heart Events and All-Cause Mortality Following Myocardial Infarction. J. Am. Coll. Cardiol..

[8] Bytyçi I., Penson P.E., Mikhailidis D.P., Wong N.D., Hernandez A.V., Sahebkar A., Thomson P., Mazidi M., Rysz J., Pella D. The prevalence of statin intolerance worldwide: A systematic review and meta-analysis with 4,143,517 patients. Proceedings of the European Society of Cardiology Congress.

[9] Banach M., Patti A.M., Giglio R.V., Cicero A.F., Atanasov A., Bajraktari G., Bruckert E., Descamps O., Djuric D.M., Ezhov M. (2018). The role of nutraceuticals in statin intolerant patients. J. Am. Coll. Cardiol..

[10] Banach M., Rizzo M., Toth P.P., Farnier M., Davidson M.H., Al-Rasadi K., Aronow W.S., Athyros V., Djuric D.M., Ezhov M.V. (2015). Statin intolerance—An attempt at a unified definition. Position paper from an International Lipid Expert Panel. Expert Opin. Drug Saf..

[11] Penson P., Toth P., Mikhailidis D., Ezhov M., Fras Z., Mitchenko O., Pella D., Sahebkar A., Rysz J., Reiner Z. (2019). P705Step by step diagnosis and management of statin intolerance: Position paper from an international lipid expert panel. Eur. Hear. J..

[12] Penson P.E., Mancini G.B.J., Toth P.P., Martin S.S., Watts G.F., Sahebkar A., Mikhailidis D.P., Banach M., on behalf of Lipid and Blood Pressure Meta-Analysis Collaboration (LBPMC) Group & International Lipid Expert Panel (ILEP) (2018). Introducing the ‘drucebo’ effect in statin therapy: A systematic review of studies comparing reported rates of statin-associated muscle symptoms, under blinded and open-label conditions. J. Cachex-Sarcopenia Muscle.

[13] Banach M., Penson P.E. (2021). Drucebo effect—The challenge we should all definitely face!. Arch. Med Sci..

[14] Penson P., Banach M. (2021). Nocebo/drucebo effect in statin-intolerant patients: An attempt at recommendations. Eur. Hear. J..

[15] Bytyci I., Bajraktari G., Sahebkar A., Penson P., Rysz J., Banach M. (2021). The prevalence of statin intolerance worldwide: A systematic review and meta-analysis with 4,143,517 patients. Eur. Heart J..

[16] Borén J., Chapman M.J., Krauss R.M., Packard C.J., Bentzon J.F., Binder C.J., Daemen M.J., Demer L.L., Hegele R.A., Nicholls S.J. (2020). Low-density lipoproteins cause atherosclerotic cardiovascular disease: Pathophysiological, genetic, and therapeutic insights: A consensus statement from the European Atherosclerosis Society Consensus Panel. Eur. Heart J..

[17] Banach M., Penson P. (2019). What have we learned about lipids and cardiovascular risk from PCSK9 inhibitor outcome trials: ODYSSEY and FOURIER?. Cardiovasc. Res..

[18] Ray K.K., Landmesser U., Leiter L.A., Kallend D., Dufour R., Karakas M., Hall T., Troquay R.P., Turner T., Visseren F. (2017). Inclisiran in patients at high cardiovascular risk with elevated LDL cholesterol. New Engl. J. Med..

[19] Dyrbuś K., Gąsior M., Penson P., Ray K.K., Banach M. (2020). Inclisiran—New hope in the management of lipid disorders?. J. Clin. Lipidol..

[20] Wright R.S., Ray K.K., Raal F.J., Kallend D.G., Jaros M., Koenig W., Leiter L.A., Landmesser U., Schwartz G.G., Friedman A. (2021). Pooled patient-level analysis of inclisiran trials in patients with familial hypercholesterolemia or atherosclerosis. J. Am. Coll. Cardiol..

[21] Henney N.C., Banach M., Penson P.E. (2021). RNA Silencing in the management of dyslipidemias. Curr. Atheroscler. Rep..

[22] Momtazi A.A., Jaafari M.R., Badiee A., Banach M., Sahebkar A. (2019). Therapeutic effect of nanoliposomal PCSK9 vaccine in a mouse model of atherosclerosis. BMC Med..

[23] Sahebkar A., Momtazi-Borojeni A.A., Banach M. (2021). PCSK9 vaccine: So near, yet so far!. Eur. Hear. J..

[24] Momtazi-Borojeni A.A., Jaafari M.R., Afshar M., Banach M., Sahebkar A. (2021). PCSK9 immunization using nanoliposomes: Preventive efficacy against hypercholesterolemia and atherosclerosis. Arch. Med Sci..

[25] Banach M., Rizzo M., Obradovic M., Montalto G., Rysz J., Mikhailidis D.P., Isenovic E.R. (2013). PCSK9 Inhibition—A novel mechanism to treat lipid disorders?. Curr. Pharm. Des..

[26] Banerjee Y., Stoian A.P., Cicero A.F.G., Fogacci F., Nikolic D., Sachinidis A., Rizvi A.A., Janez A., Rizzo M. (2021). Inclisiran: A small interfering RNA strategy targeting PCSK9 to treat hypercholesterolemia. Expert Opin. Drug Saf..

[27] Banerjee Y., Santos R.D., Al-Rasadi K., Rizzo M. (2016). Targeting PCSK9 for therapeutic gains: Have we addressed all the concerns?. Atherosclerosis.

[28] Abifadel M., Rabès J.-P., Devillers M., Munnich A., Erlich D., Junien C., Varret M., Boileau C. (2009). Mutations and polymorphisms in the proprotein convertase subtilisin kexin 9 (PCSK9) gene in cholesterol metabolism and disease. Hum. Mutat..

[29] Dewey F.E., Gusarova V., Dunbar R., O’Dushlaine C., Schurmann C., Gottesman O., McCarthy S., van Hout C.V., Bruse S., Dansky H.M. (2017). Genetic and pharmacologic inactivation of ANGPTL3 and cardiovascular Disease. N. Engl. J. Med..

[30] Crosby J.H., Peloso G.M., Auer P.L., Crosslin D.R., Stitziel N., Lange A.L., Lu Y., Tang Z.Z., Zhang H. (2014). Loss-of-function mutations in APOC3, triglycerides, and coronary disease. N. Engl. J. Med..

[31] Musunuru K., Chadwick A.C., Mizoguchi T., Garcia S.P., DeNizio J.E., Reiss C.W., Wang K., Iyer S., Dutta C., Clendaniel V. (2021). In vivo CRISPR base editing of PCSK9 durably lowers cholesterol in primates. Nature.

[32] Clark D.P., Pazdernik N.J., McGehee M.R., Clark D.P., Pazdernik N.J., McGehee M.R. (2019). Genome defense. Molecular Biology.

[33] Hille F., Richter H., Wong S.P., Bratovič M., Ressel S., Charpentier E. (2018). The biology of CRISPR-Cas: Backward and forward. Cell.

[34] Lee H., Dhingra Y., Sashital D.G. (2019). The Cas4-Cas1-Cas2 complex mediates precise prespacer processing during CRISPR adaptation. eLife.

[35] Shiimori M., Garrett S.C., Graveley B.R., Terns M.P. (2018). Cas4 nucleases define the PAM, length, and orientation of DNA fragments integrated at CRISPR Loci. Mol. Cell.

[36] Mir A., Edraki A., Lee J., Sontheimer E.J. (2017). Type II-C CRISPR-Cas9 biology, mechanism, and application. ACS Chem. Biol..

[37] Makarova K.S., Koonin E.V. (2015). Annotation and classification of crispr-cas systems. Methods Mol. Biol..

[38] Gleditzsch D., Pausch P., Esparza H.M., Özcan A., Guo X., Bange G., Randau L. (2018). PAM identification by CRISPR-Cas effector complexes: Diversified mechanisms and structures. RNA Biol..

[39] Rath D., Amlinger L., Rath A., Lundgren M. (2015). The CRISPR-Cas immune system: Biology, mechanisms and applications. Biochimie.

[40] Karginov F.V., Hannon G.J. (2010). The CRISPR system: Small RNA-guided defense in bacteria and archaea. Mol. Cell.

[41] Grainy J., Garrett S., Graveley B.R., Terns M.P. (2019). CRISPR repeat sequences and relative spacing specify DNA integration by Pyrococcus furiosus Cas1 and Cas2. Nucleic Acids Res..

[42] Richter C., Chang J.T., Fineran P.C. (2012). Function and regulation of clustered regularly interspaced short palindromic repeats (CRISPR) / CRISPR associated (Cas) systems. Viruses.

[43] Deltcheva E., Chylinski K., Sharma C.M., Gonzales K., Chao Y., Pirzada Z.A., Eckert M.R., Vogel J., Charpentier E. (2011). CRISPR RNA maturation by trans-encoded small RNA and host factor RNase III. Nature.

[44] Rutkauskas M., Krivoy A., Szczelkun M., Rouillon C., Seidel R. (2017). Single-Molecule Insight Into Target Recognition by CRISPR–Cas Complexes. Methods Enzymol..

[45] Gupta R.M., Musunuru K. (2014). Expanding the genetic editing tool kit: ZFNs, TALENs, and CRISPR-Cas9. J. Clin. Investig..

[46] Hassan M., Zhang Y., Yuan G., De K., Chen J.-G., Muchero W., Tuskan G.A., Qi Y., Yang X. (2021). Construct design for CRISPR/Cas-based genome editing in plants. Trends Plant Sci..

[47] Hu P., Zhao X., Zhang Q., Li W., Zu Y. (2018). Comparison of various nuclear localization signal-fused Cas9 proteins and Cas9 mRNA for genome editing in zebrafish. G3 Genes Genomes Genet..

[48] Jiang F., Doudna J.A. (2017). CRISPR–Cas9 Structures and mechanisms. Annu. Rev. Biophys..

[49] Lino C.A., Harper J.C., Carney J.P., Timlin J.A. (2018). Delivering CRISPR: A review of the challenges and approaches. Drug Deliv..

[50] Liu M., Rehman S., Tang X., Gu K., Fan Q., Chen D., Ma W. (2019). Methodologies for Improving HDR Efficiency. Front. Genet..

[51] Zheng T., Hou Y., Zhang P., Zhang Z., Xu Y., Zhang L., Niu L., Yang Y., Liang D., Yi F. (2017). Profiling single-guide RNA specificity reveals a mismatch sensitive core sequence. Sci. Rep..

[52] Tsai S.Q., Joung J.K. (2016). Defining and improving the genome-wide specificities of CRISPR–Cas9 nucleases. Nat. Rev. Genet..

[53] Callaway E., Ledford H. (2020). Pioneers of revolutionary CRISPR gene editing win chemistry Nobel. Nature.

[54] Dai W.-J., Zhu L.-Y., Yan Z.-Y., Xu Y., Wang Q.-L., Lu X.-J. (2016). CRISPR-Cas9 for in vivo gene therapy: Promise and hurdles. Mol. Ther. Nucleic Acids.

[55] Behr M., Zhou J., Xu B., Zhang H. (2021). In vivo delivery of CRISPR-Cas9 therapeutics: Progress and challenges. Acta Pharm. Sin. B.

[56] Wilbie D., Walther J., Mastrobattista E. (2019). Delivery aspects of CRISPR/Cas for in vivo genome editing. Accounts Chem. Res..

[57] Nishimasu H., Ran F.A., Hsu P.D., Konermann S., Shehata S.I., Dohmae N., Ishitani R., Zhang F., Nureki O. (2014). Crystal Structure of Cas9 in complex with guide RNA and target DNA. Cell.

[58] Xu C.L., Ruan M.Z.C., Mahajan V.B., Tsang S.H. (2019). Viral Delivery Systems for CRISPR. Viruses.

[59] Ledford H. (2020). CRISPR treatment inserted directly into the body for first time. Nature.

[60] Janik E., Niemcewicz M., Ceremuga M., Krzowski L., Saluk-Bijak J., Bijak M. (2020). Various Aspects of a Gene Editing System—CRISPR–Cas9. Int. J. Mol. Sci..

[61] Xie N., Zhou Y., Sun Q., Tang B. (2018). Novel epigenetic techniques provided by the CRISPR/Cas9 System. Stem Cells Int..

[62] Uddin F., Rudin C.M., Sen T. (2020). CRISPR gene therapy: Applications, limitations, and implications for the future. Front. Oncol..

[63] Wang L., Breton C., Warzecha C.C., Bell P., Yan H., He Z., White J., Zhu Y., Li M., Buza E.L. (2021). Long-term stable reduction of low-density lipoprotein in nonhuman primates following in vivo genome editing of PCSK9. Mol. Ther..

[64] Finn J.D., Smith A.R., Patel M.C., Shaw L., Youniss M.R., van Heteren J., Dirstine T., Ciullo C., Lescarbeau R., Seitzer J. (2018). A single administration of CRISPR/Cas9 lipid nanoparticles achieves robust and persistent in vivo genome editing. Cell Rep..

[65] Guo M., Xu Y., Dong Z., Zhou Z., Cong N., Gao M., Huang W., Wang Y., Liu G., Xian X. (2020). Inactivation of ApoC3 by CRISPR/Cas9 protects against atherosclerosis in hamsters. Circ. Res..

[66] Banach M., Penson P.E. (2020). Statins and LDL-C in secondary prevention—So much progress, so far to go. JAMA Netw. Open.

[67] Cybulska B., Kłosiewicz-Latoszek L., Penson P.E., Banach M. (2020). What do we know about the role of lipoprotein(a) in atherogenesis 57 years after its discovery?. Prog. Cardiovasc. Dis..

[68] Dyrbuś K., Gąsior M., Desperak P., Trzeciak P., Nowak J., Penson P.E., Osadnik T., Banach M. (2021). Risk-factors associated with extremely high cardiovascular risk of mid- and long-term mortality following myocardial infarction: Analysis of the hyperlipidaemia therapy in tertiary cardiological center (TERCET) registry. Atherosclerosis.

[69] Dyrbuś K., Gąsior M., E Penson P., Banach M. (2021). Extreme cardiovascular risk—do we need a new risk category?. Eur. Hear. J..

[70] Waghulde H., Cheng X., Galla S., Mell B., Cai J., Pruett-Miller S.M., Vazquez G., Patterson A., Kumar M.V., Joe B. (2018). Attenuation of microbiotal dysbiosis and hypertension in a CRISPR/Cas9 gene ablation rat model of GPER1. Hypertension.

[71] Limpitikul W.B., Dick I., Tester D.J., Boczek N.J., Limphong P., Yang W., Choi M.H., Babich J., DiSilvestre D., Kanter R.J. (2017). A precision medicine approach to the rescue of function on malignant calmodulinopathic long-QT syndrome. Circ. Res..

[72] Xie C., Zhang Y.-P., Song L., Luo J., Qi W., Hu J., Lu D., Yang Z., Zhang J., Xiao J. (2016). Genome editing with CRISPR/Cas9 in postnatal mice corrects PRKAG2 cardiac syndrome. Cell Res..

[73] Furgurson M., Lagor W.R. (2019). CRISPR. Curr. Opin. Lipidol..

[74] Zhao Y., Yang Y., Xing R., Cui X., Xiao Y., Xie L., You P., Wang T., Zeng L., Peng W. (2018). Hyperlipidemia induces typical atherosclerosis development in Ldlr and Apoe deficient rats. Atherosclerosis.

[75] Huang L., Hua Z., Xiao H., Cheng Y., Xu K., Gao Q., Xia Y., Liu Y., Zhang X., Zheng X. (2017). CRISPR/Cas9-mediated ApoE-/- and LDLR-/- double gene knockout in pigs elevates serum LDL-C and TC levels. Oncotarget.

